# Distance to thrombus, ischemic lesion volume and clinical outcome after thrombectomy for M1 middle cerebral artery occlusion

**DOI:** 10.1007/s00508-024-02364-y

**Published:** 2024-05-15

**Authors:** Katharina Millesi, Monika Killer-Oberpfalzer, Johannes A. R. Pfaff, J. Sebastian Mutzenbach, Christoph J. Griessenauer, Michael Sonnberger, Milan Vosko, Judith Wagner, Matthias Millesi, Slaven Pikija, Constantin Hecker

**Affiliations:** 1 Department of Neurology, Paracelsus Medical University Salzburg, Salzburg, Austria; 2 Institute of Neurointervention, Paracelsus Medical University Salzburg, Ignaz-Harrer-Straße 79, 5020 Salzburg, Austria; 3 Department of Neuroradiology, Paracelsus Medical University Salzburg, Salzburg, Austria; 4 Department of Neurosurgery, Paracelsus Medical University Salzburg, Salzburg, Austria; 5 Department of Neuroradiology, Neuromed Campus, Johannes Kepler University, Linz, Austria; 6 Department of Neurology 2, Med Campus III, Kepler University Hospital, Johannes Kepler University, Linz, Austria; 7 Department of Neurology, Evangelisches Klinikum Gelsenkirchen, Teaching Hospital University Duisburg-Essen, Gelsenkirchen, Germany; 8 Department of Neurosurgery, Medical University of Vienna, Vienna, Austria

**Keywords:** Stroke, Large vessel occlusion, Neurointervention, Outcome, Thrombus

## Abstract

**Background:**

Stroke resulting from occlusion of the middle cerebral artery (MCA) can have devastating consequences, potentially leading to a loss of independence. This study aimed to investigate the relationship between the distance to the thrombus (DT) and both ischemic lesion volume (ILV) and clinical outcomes.

**Methods:**

We retrospectively evaluated patients with thromboembolic MCA M1 segment occlusion who underwent neurovascular imaging followed by endovascular thrombectomy (EVT) at two comprehensive stroke centers over a 3-year period (2018–2020). Preinterventional computed tomography (CT) or magnetic resonance (MR) angiography was used to measure DT, defined as the distance from the carotid‑T bifurcation to the proximal surface of the M1 occlusion. Postinterventional CT or MR imaging was employed to determine the ILV and clinical outcomes were assessed using the modified Rankin scale (mRS) at 3 months.

**Results:**

There were 346 patients evaluated. The median DT was 9.4 mm (interquartile range, IQR 6.0–13.7 mm) and the median ILV was 13.9 ml (IQR 2.2–53.1 ml). After adjustment, an increase in DT was associated with a decrease in odds for a larger ILV (odds ratio, OR 0.96, 95% confidence interval, CI 0.92–0.99, *p* = 0.041). Through this association, more distal thrombi were associated with good clinical outcome (mRS 0–2; clinical outcome available in 282 patients, *p* = 0.018). The ILV was inversely associated with better clinical outcome OR 0.52 (95% CI 0.40–0.67).

**Conclusion:**

Based on the findings, DT was identified as an independent albeit weak predictor for ILV and clinical outcomes in patients with MCA M1 occlusion who underwent EVT.

**Supplementary Information:**

The online version of this article (10.1007/s00508-024-02364-y) contains supplementary material, which is available to authorized users.

## Introduction

Stroke resulting from large vessel occlusion (LVO) of the proximal middle cerebral artery (MCA) is a devastating disease and a significant contributor to loss of independence [1]. Endovascular thrombectomy (EVT) as a reperfusion procedure for removal of the occluding thrombus is a mainstay of treatment [2]. In this sense, sufficient recanalization is associated with better clinical outcome; however, sufficient reperfusion cannot be achieved in all patients [3]. Unfortunately, even after successful recanalization, in a number of patients the clinical outcome remains poor [4, 5].

Although successful recanalization is a primary driver for a better outcome, many other factors influence the clinical course. The distance to the thrombus (DT) as measured from the T‑bifurcation of the internal carotid artery (ICA) to the proximal thrombus surface, can be readily determined and was shown to be of prognostic value [6]. Although a longer DT was associated with better clinical outcomes, it was not consistently associated with recanalization success [7]. The postinterventional ischemic lesion (i.e., infarct) volume was shown to correlate with functional outcome in patients treated with EVT, even when sampled early (within 24 h) after onset [8, 9].

We aimed to characterize the DT and its relationship with neuroradiological outcome measured as ischemic lesion volume 24 h after EVT, in the contemporary sample of patients treated at two comprehensive stroke centers presenting due to emergent MCA M1 occlusion.

## Methods

### Study population

For this study all patients admitted for acute ischemic stroke due to LVO at two comprehensive stroke centers from 1 January 2018 to 31 December 2020 coming within 24 h since last-seen-well and without contraindications to mechanical thrombectomy were eligible. Specifically, we excluded patients with a modified Rankin scale (mRS) greater than 3 prior to presentation. Treatment with intravenous alteplase was allowed. For definitive study inclusion, performance of neurovascular imaging by either CT or MR angiography (CTA or MRA) followed by EVT due to an occlusion of the M1 segment of the MCA (defined as thrombus material situated between the T‑bifurcation of the ICA and the MCA bifurcation) was required. Additionally, individual comorbidities such a history of prior stroke, atrial fibrillation and peripheral arterial occlusive disease in each patient, together with prestroke medication (e.g., intake of anticoagulants) were documented. Devices used for the EVT technique (i.e., thrombus aspiration alone vs. pass with stent retriever alone vs. combination of both), procedural times, complications, and the type of anesthesia was noted. At admission, stroke severity, classified by the National Institutes of Health stroke scale (NIHSS), was evaluated by a certified stroke physician. Due to the study’s retrospective nature, the ethics committee’s approval was waived. The measurements were performed by a neurologist with > 20 years experience in vascular neurology (SP) and by a second experienced neurologist (> 10 years experience) to perform interrater variability.

### Evaluation of neurovascular imaging

**Fig. 1:**
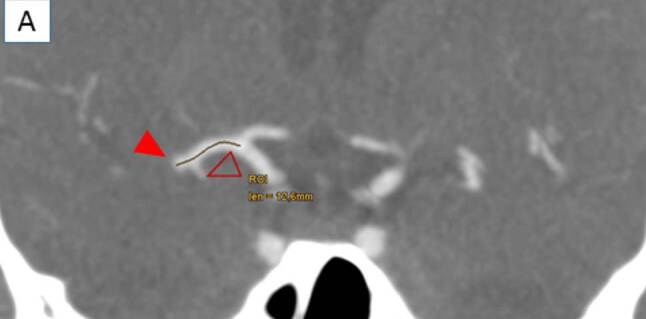
Depiction of the measurement of the distance to thrombus (DT) from the middle of the T-Bifurcation of the internal carotid artery (empty arrow) to the cessation of contrast medium as the proximal end of the thrombus (filled arrow) based on a CT-angiography (12.6 mm in this example)

The CTA or MRA performed in each patient needed to include the depiction of the arterial circle of Willis together with adequate native image resolution for evaluation of the ASPECTS score and leptomeningeal collaterals (LC) [10]. The presence and the quality of the observed LC were then classified as being either “good” or “poor,” as described previously [11]. In this sense, a situation of “good” collaterals was present if the collateral filling was equal as opposed to “poor” collaterals if the filling was less than the other hemisphere or no filling [11]. The DT was then measured manually with the Deep Unity® curve tool (Dedalus HealthCare, Bonn, Germany) (in mm) from the middle of the T‑bifurcation of the internal carotid artery to the cessation of contrast medium as the proximal end of the thrombus as suggested by Friedrich et al. [6] (see Fig. 1). The DT was additionally divided into equally numbered groups with progressive distance and designated “proximal” (0.0–7.2 mm), “middle” (7.3–12.1 mm) and “distal” (12.2–27.8 mm).

### Characterization of radiological outcome

The outcome of the EVT was categorized according to the modified thrombolysis in cerebral infarction (mTICI) score ranging from 0 to 3. These were grouped as insufficient reperfusion (0–2a) or sufficient reperfusion (2b, 3). The volume of the ischemic lesion volume (i.e., infarction) in each patient was calculated 24 h after performing EVT and according to the ABC/2 formula [12] and documented (in ml). Virtually all patients had CT scans as control imaging. Edema and/or hemorrhagic transformation were included in the ILV measurement. The signs of evidently old infarctions in the ipsilateral hemisphere were neglected and best efforts were made to exclude possible gliosis associated with old infarcts in the vicinity of a new infarction.

### Characterization of clinical outcome

Clinical outcome was measured by a modified Rankin scale at 3 months. The patients were examined at 3 months by a clinician or interviewed via telephone (the caregiver would be questioned when the patient could not provide information). In the event of death, the data were sampled from the relevant municipal registries.

### Statistical analysis

Categorical variables were prepared as absolute numbers and percentages, whereas continuous variables were tested (Kolmogorov-Smirnov test) for normal distribution. Non-normally distributed data were presented as the median and interquartile range (IQR, 25–75 percentile). As data were non-normally distributed, non-parametric tests of associations (Wilcoxon Rank-Sum test, Spearman Rho for correlation between continuous variables) were performed. Exploration analysis was performed with ln-transformed ILV, and ILV was categorized into four categories (0–15 ml, 15.1–70 ml, 70.1–200 ml, and > 200 ml) as the dependent variable [9]. First, we performed univariate non-parametric analysis (Kruskal-Wallis test for binary-continuous, and Fisher’s test with simulated *p* values, when needed, for categorical variables) for possible associations. The ILV and ln-transformed ILV was also inspected as a continuous predictor. All predictors having *p* < 0.1 entered the multivariate analysis. In multivariate ordinal regression analysis, where ILV was grouped into the previously described categories, the following adjusting variables were accounted for: (1) continuous predictors: age in years at symptom onset, total thrombectomy steps performed, distance to thrombus in mm, (2) categorical variables: the technique used for thrombus removal (aspiration + stent retriever vs. aspiration only, vs. stent retriever only) as a binary variable state of the leptomeningeal collaterals as seen on first imaging grouped into good/equal vs. bad; ASPECT score greater than 6, TICI outcome 2b–3 vs. 0–2a and intravenous thrombolysis. Missing data were managed with multivariate imputation via chained equation (MICE) package in R. We also performed univariate mediation analysis, adjusted for age (in years), TICI outcome (0-2a vs. 2b–3, as binary variable), ASPECTS score greater than 6 and thrombolysis (binary), where DT was considered a predictor, ln-transformed ILV as a mediator, and mRS at 3 months (0–2 vs. 3–6) as outcome. The interrater reliability was determined through an intraclass correlation calculation on 19 randomly selected patients. The level of statistical significance was set at *P* < 0.05. For statistical analyses, we used R Software v. 4.2.0 [13].

## Results

### Study population

Overall, 353 patients underwent endovascular thrombectomy in the anterior circulation for LVO at both participating centers, and 346 fulfilled all criteria for inclusion in this study. The median age was 76 years (IQR 64–83 years), and the proportion of women was 58%. The median NIHSS at admission was 16 (IQR 12–19), and the proportion of wake-up stroke was 20%. A known history of previous stroke or TIA was present in 37 patients (11%). Intravenous thrombolysis with recombinant tissue plasminogen activator (rt-PA) (IVT) was administered in 187 (54%) patients. Median time from end of EVT and first control CT imaging was 1 day (IQR 1–1 day). Detailed demographic characteristics are listed in Table 1 and information about missing data is given in supplemental Table 1.

**Table 1:** Final infarct volume in 346 patients treated with thrombectomy for emergent middle cerebral artery M1 segment occlusion at 2 centers in a 3‑year period (2018–2020)

		Ischemic lesion volume (in ml)				
Variables	All patients, N = 346 (%)	0–15, N = 181 (%)	15.1–70, N = 96 (%)	70.1–200, N = 45 (%)	> 200, N = 24 (%)	P value
**Demographics**						
*Study site*	–	–	–	–	–	0.053
Linz	235 (68)	122 (67)	73 (76)	24 (53)	16 (67)	–
Salzburg	111 (32)	59 (33)	23 (24)	21 (47)	8 (33)	–
*Age (years)*	76 (64–83)	77 (69–84)	71 (61–82)	78 (71–83)	66 (54–80)	0.013
*Female*	200 (58)	113 (62)	49 (51)	27 (60)	12 (50)	0.277
*NIHSS at admission*	16 (12–19)	16 (12–19)	16 (12–18)	17 (12–20)	18 (15–21)	0.131
*Wake-up stroke*	70 (20)	25 (14)	26 (27)	13 (29)	6 (25)	0.020
*Previous stroke/TIA*	37 (11)	19 (10)	13 (14)	5 (11)	0 (0)	0.260
*Peripheral arterial occlusive disease*	15 (4)	9 (5)	3 (3)	1 (2)	2 (8)	0.534
*Atrial fibrilation*	113 (33)	63 (35)	28 (29)	14 (31)	8 (33)	0.806
*Diabetes*	35 (10)	18 (10)	10 (10)	5 (11)	2 (8)	0.991
*Arterial hypertension*	176 (51)	93 (51)	48 (50)	23 (51)	12 (50)	0.998
*ICA stenosis ≥* *50%*	37 (11)	20 (11)	12 (12)	3 (7)	2 (8)	0.820
*Angina pectoris*	50 (14)	29 (16)	14 (15)	5 (11)	2 (8)	0.775
*Taking antithrombotic agents*	53 (15)	33 (18)	13 (14)	5 (11)	2 (8)	0.504
*Taking vitamin K anticoagulants*	16 (5)	8 (4)	4 (4)	1 (2)	3 (12)	0.297
*Taking non-vitamin K anticoagulants*	35 (10)	16 (9)	9 (9)	9 (20)	1 (4)	0.140
*TOAST*	–	–	–	–	–	0.609
cardioembolic	225 (66)	125 (69)	59 (63)	25 (57)	16 (67)	–
large-artery atherosclerosis	43 (13)	22 (12)	14 (15)	6 (14)	1 (4)	–
other etiology	6 (2)	3 (2)	2 (2)	0 (0)	1 (4)	–
undetermined	2 (1)	1 (1)	1 (1)	0 (0)	0 (0)	–
unknown	67 (20)	30 (17)	18 (19)	13 (30)	6 (25)	–
**Neuroimaging data**						
*MCA M1 right side*	176 (51)	90 (50)	47 (49)	24 (53)	15 (62)	0.650
*ASPECTS*	–	–	–	–	–	< 0.001
≤ 6	42 (12)	8 (4)	15 (16)	11 (25)	8 (33)	–
> 6	298 (87)	171 (95)	78 (84)	33 (75)	16 (67)	–
*Good or equal leptomeningeal collaterals*	191 (56)	110 (60)	49 (53)	24 (55)	8 (33)	0.082
*Distance to thrombus (mm)*	9 (6–14)	10 (7–14)	9 (6–14)	8 (5–11)	8 (6–15)	0.047
*Distance to thrombus (categorized)*	–	–	–	–	–	0.089
Proximal	115 (33)	48 (27)	36 (38)	22 (49)	9 (38)	–
Middle	112 (32)	67 (37)	26 (27)	13 (29)	6 (25)	–
Distal	119 (34)	66 (36)	34 (35)	10 (22)	9 (38)	–
**Intervention data**						
*Systemic thrombolysis*	187 (54)	105 (58)	51 (53)	18 (40)	13 (54)	0.191
*Type of anesthesia*	–	–	–	–	–	0.263
CS	25 (7)	18 (10)	2 (2)	4 (9)	1 (4)	–
CS then GA	2 (1)	1 (1)	1 (1)	0 (0)	0 (0)	–
GA	317 (92)	160 (89)	93 (97)	41 (91)	23 (96)	–
Local	1 (0)	1 (1)	0 (0)	0 (0)	0 (0)	–
**Chronometry**						
*Time from groin puncture to target vessel (min)*	8 (5–15)	8 (5–15)	7 (5–13)	8 (5–21)	10 (6–16)	0.519
*Thrombolysis to groin puncture (min)*	102 (49–128)	92 (44–125)	114 (86–138)	108 (35–128)	90 (70–122)	0.181
*First imaging to vessel puncture (min)*	130 (71–160)	127 (65–156)	142 (108–179)	114 (50–154)	131 (99–154)	0.026
*CT imaging to target vessel (min)*	140 (86–170)	132 (80–170)	149 (116–188)	124 (67–161)	142 (106–166)	0.038
*Procedure time (min)*	40 (23–70)	35 (21–64)	42 (21–65)	67 (36–90)	46 (35–74)	< 0.001
**Procedural data**						
*Type of device used*	–	–	–	–	–	0.032
aspiration + stent retriever	131 (42)	56 (35)	38 (42)	26 (65)	11 (50)	–
aspiration only	121 (39)	73 (45)	31 (34)	10 (25)	7 (32)	–
stent retriever only	62 (20)	33 (20)	21 (23)	4 (10)	4 (18)	–
*Total thrombectomy steps performed*	1 (1–3)	1 (1–2)	1 (1–2)	2 (2–4)	3 (2–4)	< 0.001
*First pass successful*	163 (49)	99 (56)	48 (51)	10 (23)	6 (26)	< 0.001
*TICI outcome*	–	–	–	–	–	< 0.001
0–2a	32 (9)	5 (3)	6 (6)	14 (31)	7 (29)	–
2b–3	313 (91)	175 (97)	90 (94)	31 (69)	17 (71)	–
2b vs. 3	–	–	–	–	–	0.462
2b	52 (15)	23 (13)	19 (20)	7 (16)	3 (12)	–
3	293 (85)	157 (87)	77 (80)	38 (84)	21 (88)	–
**ICH**						
Presence of ICH	125 (36)	41 (23)	43 (45)	26 (58)	15 (62)	< 0.001
Symptomatic ICH	14 (4)	2 (1)	4 (4)	6 (13)	2 (8)	< 0.001

Data are counts (%) and median (interquartile range). Data for type of device used are missing for 32 (9.2%) of patients. TICI outcome missing for 1 (0.2%) patient.

*ASPECTS* Alberta Stroke Program Early CT Score, *CS* conscious sedation, *GA* general anesthesia, *ICA* internal carotid artery, *ICH* intracranial hemorrhage, *MCA* middle cerebral artery, *NIHSS* National Institutes of Health Stroke Scale, *TIA* transient ischemic attack, *TICI* thrombolysis in cerebral infarction score, *TOAST* Trial of Org 10172 in Acute Stroke Treatment classification

### Distance to thrombus

**Fig. 2:**
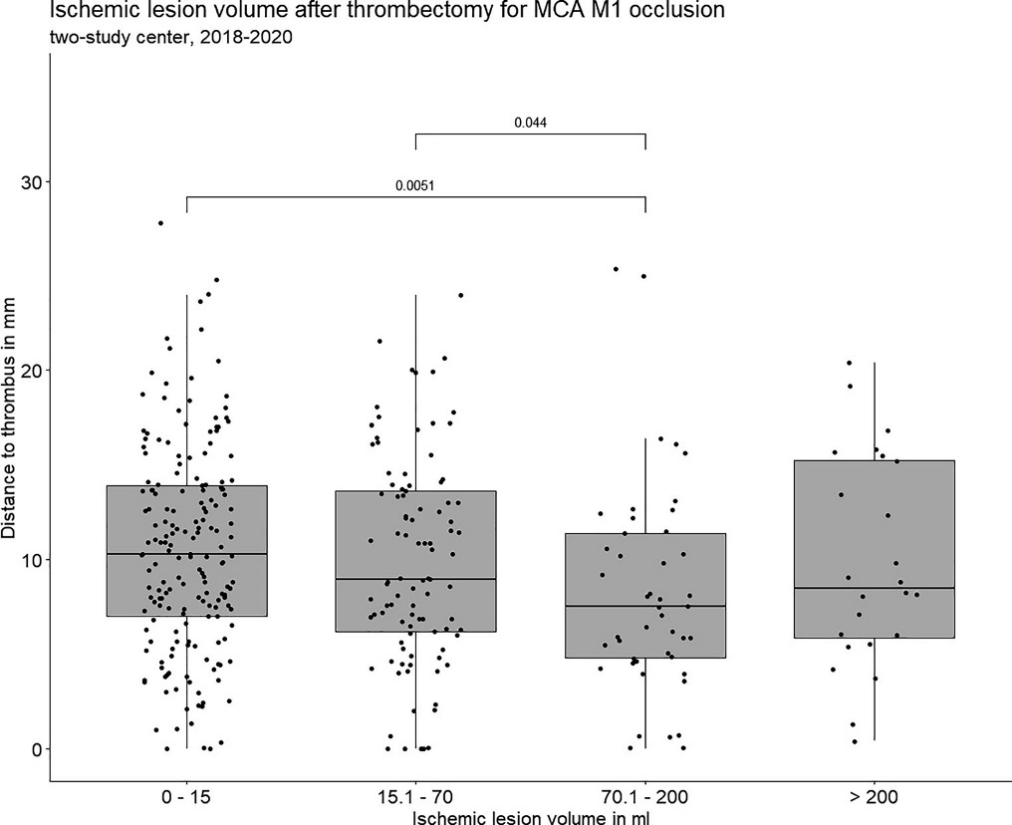
Ischemic lesion volume (in ml) in relation to distance to thrombus (in mm) after mechanical thrombectomy in 346 patients with middle cerebral artery M1 occlusion treated during three year period 2018-2020 at two centers

The median DT was 9mm (IQR 6–14mm) and was progressively smaller across larger ILV, except for the largest ILV, where no difference between other ILV groups was found (Fig. 2). A larger DT was associated with smaller ILV (ln-transformed), although the effect was small, r (344) = −0.15, *p* = 0.005 (Supplemental Fig. 1). Additionally, after splitting DT into three equal groups (range of values in mm: 0–7, > 7–12, > 12–28 for proximal, middle, and distal groups, respectively), the association with continuous ILV was significant at *p* = 0.003. Similarly, continuous DT was significantly associated with the ILV groups 0–15 ml vs. 70.1–200 ml and 15.1–70 ml vs. 70.1–200 ml, at *p* = 0.047. The measurements made by the two raters, who both have over 5 years of experience in the neurovascular field, demonstrated a high level of agreement in determining the DT on CT/MR images with an intraclass correlation coefficient of 0.87 (95% CI 0.69–0.95) for DT on CTA/MRA images.

The median ILV was 13.9 ml (IQR 2.2–53.1 ml). Age was significantly, albeit complexly, associated with ILV. The group with the largest ILV was the youngest; generally, the age had a negative correlation with ILV, r (345) = −0.13, *p* = 0.013. Patients waking up with stroke symptoms had significantly larger ILV (40.8 ml vs. 11.1 ml, *p* < 0.001). A lower ASPECTS score was predictive of a larger ILV (51.0 ml vs. 11.0 ml, *p* < 0.001). Good LC was protective for larger ILV (equal group having 5.8 ml vs. absent group having 17.3 ml, *p* = 0.021). Procedure time was the shortest in the smallest ILV group (35 min vs. 67 min. for 0–15 ml vs. 70.1–200 ml group, respectively, *p* < 0.001). Both devices used were associated with the largest ILV (13.9 ml vs. 8.7 ml for aspiration + stent retriever vs. aspiration only, *p* = 0.021). Total EVT passes performed were also positively correlated with ILV, 3 (IQR 2-4) steps in the largest ILV group, *p* < 0.001. Unsuccessful EVT measured by mTICI was associated with larger ILV (96.2 ml vs. 11.5 ml for 0–2a vs. 2b–3 mTICI outcome, respectively, *p* < 0.001) (Table 2). With an intraclass correlation coefficient 0.78 (CI 0.54–0.91) for ischemic infarct volume, good agreement in determining the ischemic lesion volume was also shown.

**Table 2:** Correlations of various variables with final infarct volume on continuous scale in 346 patients treated with thrombectomy for emergent middle cerebral artery M1 segment occlusion at 2 centers in a 3‑year period (2018–2020)

Variable	Median (IQR)	Rho corr. coef	P value
*Age (years)*	–	−0.12	0.029
*NIHSS at admission*	–	0.15	0.007
*Wake-up stroke (yes)*	13.9 (2.2–53.0)	–	< 0.001
*Leptomeningeal collaterals*	13.9 (2.2–53.1)	–	0.022
Absent	17.3 (3.8–65.0)	–	–
Good	11.4 (1.7–52.6)	–	–
Equal	5.8 (1.8–36.5)	–	–
*Systemic thrombolysis*	−0.34 (−0.75, 0.06)	–	0.10
*Symptomatic ICH*	13.9 (2.2–53.0)	–	0.002
*Total thrombectomy steps performed*	–	0.31	< 0.001
*First pass successful*	13.9 (2.2–53.0)	–	< 0.001
*Good leptomeningeal collaterals*	8.9 (1.7–46.4)	–	0.012
*TICI outcome*	13.9 (2.2–53.0)	–	< 0.001
0–2a	96.2 (38.4–177.7)	–	–
2b–3	11.5 (2.0–45.0)	–	–
*Type of device used*	13.9 (2.2–53.0)	–	0.022
Aspir + stent.retr	25.3 (3.1–87.5)	–	–
Aspir only	8.7 (1.8–31.6)	–	–
Stent.retr only	13.7 (2.6–40.7)	–	–
*ASPECTS* *≤* *6*	13.9 (2.2–53.0)	–	< 0.001
*Distance to thrombus*	13.9 (2.2–53.0)	–	0.003
Proximal	23.4 (5.2–73.0)	–	–
Middle	11.9 (2.6–34.5)	–	–
Distal	8.2 (0.6–47.2)	–	–

Data are rho coefficient, and median (interquartile range)

*ASPECTS* Alberta Stroke Program Early CT Score, *ICH* intracranial hemorrhage, *NIHSS* National Institutes of Health Stroke Scale, *TICI* thrombolysis in cerebral infarction

There were 64 (18%) missing mRS data. Therefore, the logistic regression analysis was performed on 282 patients. Good outcome (0–2 at 3 months) was recorded in 139 (49%) patients. Age (71 vs. 79 years, OR 0.96, CI 0.96–0.98, *p* < 0.001), not taking vitamin K oral anticoagulant (OAC) (1% vs. 7%, OR 0.20, CI 0.03–0.77, *p* = 0.038), thrombolysis (61% vs. 47%, OR 1.68, CI 1.05–2.70, *p* = 0.030), ASPECTS > 6 (93% vs. 83%, OR 2.58, CI 1.22–5.87, *p* = 0.017), less total thrombectomy steps performed (1 vs. 2, OR 0.82, 0.70–0.95, *p* = 0.009), successful first pass (59% vs. 52%, OR 2.44, CI 1.50–3.98, *p* < 0.001), 2b and 3 vs. 0–2a TICI outcome (94% vs. 86%, OR 0.34, CI 0.14–0.77, *p* = 0.013), lower ln-ILV (3.9 vs. 35.9, OR 0.61, CI 0.52–0.71, *p* < 0.001) were all associated with good outcome (Supplemental Table 2). The above predictors entered multivariate logistic regression analysis.

### Multivariate regression analysis

#### Ischemic lesion volume

After adjusting for variables listed in the methods, R^2^ showed that our model only modestly explains (~27%) the variance in the ordinal dependent variable ILV. The best predictive value was observed for ILV 0–15ml and 15.1–70ml at *p* = 0.001. The most significant effect on ILV was for unsuccessful EVT measured by mTICI, raising the odds for larger ILV with an OR of 7.17 (CI 3.64–14.29). Stent retriever device usage (alone or with aspiration device) heightened odds for larger ILV, with an OR of 1.55 (CI 1.01–2.40), as was ASPECTS > 6 with an OR of 0.27 (CI 0.15–0.51). Finally, the DT per mm increase lowered the odds for larger ILV, with an OR of 0.96 (CI 0.92–0.99), *p* = 0.048 (Table 3).

**Table 3:** Multivariate ordinal logistic analysis with final infarct volume (in ml) as outcome variable in 346 patients treated with thrombectomy for emergent middle cerebral artery M1 segment occlusion at 2 centers in a 3‑year period (2018–2020)

	Odds ratio	95% CI	P value
*Infarct volume in ml*			
0–15|15.1–70	0.11	0.029, 0.417	0.001
15.1–70|70.1–200	0.52	0.138, 1.931	0.324
70.1–200|> 200	2.19	0.560, 8.330	0.263
*Age in years*	0.99	0.971, 1.002	0.090
*Good or equal leptomeningeal collaterals*	0.80	0.518, 1.229	0.305
*TICI 0–2a*	7.17	3.644, 14.286	< 0.001
*Stent retriever* *±* *aspiration*	1.55	1.012, 2.402	0.046
*ASPECTS* *>* *6*	0.27	0.146, 0.512	< 0.001
*Distance to thrombus on first imaging in mm*	0.96	0.922, 0.999	0.041
*Systemic thrombolysis*	0.93	0.608, 1.424	0.737

Observations: 346; R^2^ Nagelkerke: 0.089

*ASPECTS* Alberta Stroke Program Early CT Score, *CI* confidence interval, *NIHSS* National Institutes of Health Stroke Scale, *TICI* thrombolysis in cerebral infarction

#### Clinical outcome

After adjusting for variables showing *p* < 0.1 on univariate analysis (e.g., age, NIHSS at admission, vitamin K OAC, known time of symptom onset, systemic thrombolysis, ASPECTS > 6, good or equal LC, ipsilateral ICA diameter, total thrombectomy steps performed, successful first pass, TICI 0–2a, vessel perforation, hemorrhagic transformation type, ln-transformed ILV), the following variables showed a significant and negative association with good clinical outcome at 3 months: age (OR 0.94, 95% CI 0.91–0.96, *p* < 0.001), NIHSS at admission (OR 0.87, 95% CI 0.81–0.93, *p* < 0.001), class 3a–d hemorrhagic transformation vs. class I (OR 0.16, 95% CI 0.03–0.83, *p* = 0.03), ln-transfomed ILV (OR 0.52, 95% CI 0.40–0.67, *p* < 0.001) (Supplemental Table 3).

### Mediation analysis

The mediation model in which DT was considered as a predictor, ILV a mediator, and good clinical outcome at 3 months as an outcome, indicated that the effect of distance to a thrombus (i.e., longer DT) on the clinical outcome at 3 months (mRS 0–2 vs. 3–6) was fully mediated via the ln-transformed infarct volume. The indirect effect of distance to thrombus in the mediation model was small: 0.005; however, it was statistically significant (*p* = 0.018). After adjusting for age, thrombectomy outcome, and ASPECTS (> 6 vs. ≤ 6), the mediation stayed statistically significant (*p* = 0.016); however, the effect was small at 0.004.

## Discussion

We found that distance to thrombus is associated with infarct volume in the large two-center contemporary sample of patients treated with endovascular thrombectomy for emergent MCA M1 occlusion. Through mediation analysis, more distant thrombi were also shown to be associated with better clinical outcome.

Our results could facilitate patient care since ILV correlates well with the clinical outcome [8, 9]. Furthermore, DT measurement, forgoing complex postprocessing analyses, could be readily applied, even in the acute setting.

The patient, and other stakeholders involved in their care, could profit from early reliable information about prognostication, as noted by Bucker et al., and ILV is suited for such an endeavor [8]. On the other side, ILV is known to change after insult, and cytotoxic edema, hemorrhagic transformation, or even recurrent ischemic events play a role. Indeed, ILV growth between the first imaging at 24 h and the second after 1 week is common [8]; however, there are good data to ascertain that even early CT-assessed ILV is of good prognostication value [8]. Theoretical disadvantage of relying solely on ILV as a predictor for the clinical outcome is a lack of information about the side of the brain involved (and sometimes also the sidedness of the patient). It’s commonly assumed that lesions on the left side, which typically involve speech centers, lead to poorer outcomes. However, this isn’t consistently proven across the literature [8, 14].

As Al Ajlan et al. showed in a similarly large cohort, including ICA occlusions, EVT outcome and especially unsuccessful recanalization were associated with larger ILV [14]. Other variables, such as NIHSS and ASPECTS, were also associated with ILV.

Other studies generally were ambiguous in associating DT as measured pre-angiographically with the clinical outcome. The study by Friedrich et al. showed in the 136 patients treated exclusively with thrombolysis that DT lower than 16 mm was predictive of unfavorable results [6]. Lobsien et al. found that DT was not predictive of clinical outcomes in 87 patients comparing EVT vs. thrombolysis [15]. In a group of 55 patients, Gawlitza et al. investigated the influence of DT on Tmax perfusion deficit. They found it as an independent predictor of target mismatch, concluding that lower DT (more proximal MCA occlusion) is associated with more extensive tissue at risk [16]; however, these previous studies on DT were done almost exclusively in the pre-thrombectomy era, rendering their comparability with our contemporary sample difficult. Although we did not detect a direct relationship between DT and clinical outcome, a more distant position of the thrombus (larger DT) showed a small but significant association with good clinical outcome, even after adjustments, when assessed with mediation analysis.

We learned that although DT followed the path of an inverse linear association with ILV, meaning a thrombus further from the T‑bifurcation (higher DT) leads to lower ILV, this association unexpectedly breaks for the patients with the largest ILV. Therefore, the distribution of the data follows a reversed J‑shaped distribution. The reason thereof is most probably multifaceted. One explanation could be that as we did not know where the thrombus location at the symptom onset was, we could be seeing the aftermath of thrombus passage through the MCA trunk. We are measuring the “second” position thereof. Kaesmacher et al. demonstrated this effectively in their study. They concluded that some of the thrombi had already shifted position and left infarction within lenticulostriatal arteries perfusion area before the initial imaging [17]. Thrombi are known to migrate, especially after thrombolysis, and fragment after that [18]. This is supported by our data showing that this group had the highest proportion of low ASPECTS score, most probably representing areas already infarcted. Additionally, this group had the lowest percentage of good/equal LC numerically. Finally, the patients in this group had the second-highest rate of unsuccessful EVT and the highest number of EVT steps. These data are suggestive that patients with the highest ILV were most probably having a “perfect storm” of (1) already sustained damage; (2) complex anatomy and/or difficulty in extracting thrombus material; (3) failed collateral circulation. Even with the modern devices, the extraction of distally placed thrombus could be challenging, and the number of EVT passes performed might be higher. Compared to other studies, we have large number of post-procedural hemorrhagic transformation and bleeding (36%), most probably due to methodology used—Heidelberg classification, indeed class I bleeding that also includes small petechial bleedings accounted for 22%. Symptomatic bleeding accounted for 4%, and this result is comparable to other published studies [19].

### Limitations

Although our study was conducted in the contemporary 3‑year period in 2 large comprehensive stroke centers with significant stroke patient turn-around, some limitations prevent the generalization of our conclusions. The study’s retrospective design most probably introduced biases we could not account for. We did not include all patients with unknown time of symptom onset, only those having symptoms upon awakening. Only M1 occlusions were included in this analysis and the observed results cannot be extrapolated for ICA occlusions. Also, this analysis did not assess the exact position of the thrombus in relation to the perforating arteries or their patency. We do not have the status of infarct core volume at first imaging as we did not routinely perform perfusion imaging or MRI-DWI in every patient. This reflects the real-life setting of the study. Using CT can be less sensitive for measuring ILV compared to MRI; however, at least one study showed no significant difference between both techniques [14]. Furthermore, ABC/2 volume determination has some limitations, and results could be different when automatic ILV measurement has been used. Additionally, the results could have been different if we had had MRI as control imaging. There is a lack of universal mRS documentation for our patients (18% missing); however, as already mentioned, ILV could serve as a proxy outcome measure. As we included only MCA occlusions our results are not immediately generalizable to other types of LVO.

## Conclusion

The DT was found to serve as an independent but weak predictor for ILV and clinical outcome in the event of MCA M1 occlusion in a patient cohort that underwent EVT. More studies with rigorous methodology are needed.

## Supplementary Information

Suppl. Tab. 1 Percentage of missing data from demographic table.

Suppl. Tab. 2 Univariate associations with clinical outcome at 3 months as measured with modified Rankin Scale (mRS).

Suppl. Tab. 3 Multivariate logistic regression analysis of 242 patients, good clinical outcome was defined as modified Rankin scale 0–2 at 3 months.

Suppl. Fig. 1 ln-transformed ischemic lesion volume after thrombectomy for MCA M1 occlusion
