# Supplementary material for: Distance to thrombus, ischemic lesion volume and clinical outcome after thrombectomy for M1 middle cerebral artery occlusion
Source: Wien Klin Wochenschr. 2024 May 15;137(5-6):163–71. doi: 10.1007/s00508-024-02364-y (PMC11926011; doi:10.1007/s00508-024-02364-y)
Supplement: Supplementary file 1 — Suppl. Tab. 1 Percentage of missing data from demographic table. [file 508_2024_2364_MOESM1_ESM.docx]

| **Variables** | **Miss.perc.** |
| --- | --- |
| site | 0% |
| Age (years) | 0% |
| Gender | 0% |
| NIHSS at admission | 10% |
| Wake-up stroke | 0% |
| Stroke/TIA | 0% |
| Peripheral arterial occlusive disease | 0% |
| Atrial fibrillation known prior to stroke | 0% |
| Diabetes | 0% |
| Arterial hypertension | 0% |
| Internal carotid artery stenosis ≥ 50% | 0% |
| Angina pectoris | 0% |
| Taking antithrombotics | 0% |
| Taking Vitamin-K anticoagulants | 0% |
| Taking non-vitamin K anticoagulants | 0% |
| Known kidney failure | 0% |
| Known heart failure | 0% |
| Leptomeningeal collaterals | 1% |
| Side of vessel occluded | 0% |
| Systemic thrombolysis | 0% |
| mca.length.curve | 4% |
| mca.length.straight | 5% |
| Ipsilateral ICA diameter (mm) | 1% |
| Ispilateral MCA M1 diameter (mm) | 2% |
| Ispilateral MCA M2 diameter (mm) | 5% |
| Type of anestesy | 0% |
| (CS) |  |
| (CS then GA) |  |
| (GA) |  |
| (Local) |  |
| Time from groin puncture to target vessel | 1% |
| Symptom onset to groin puncture (min) | 32% |
| Procedure time (min) | 1% |
| Vessel open in DSA | 1% |
| Onset to target vessel (min) | 69% |
| Symptomatic ICB | 0% |
| Total thrombectomy steps performed | 1% |
| Final infarction volume | 0% |
| First pass successful | 3% |
| Leptomeningeal collaterals | 1% |
| TICI outcome | 0% |
| Pre morbid mRS | 46% |
| Type of device used | 9% |
| First imaging to vessel puncture (min) | 3% |
| CT imaging to target vessel (min) | 1% |
| ASPECTS | 2% |
| Distance to thrombus on first imaging (mm) | 0% |
| Modified Rankin Scale at 3 months | 18% |

**Suplemmental Table 1.** Percentage of missing data from demographic table.

Abbreviations: ASPECTS – Alberta Stroke Program Early CT Score, CS – conscious sedation, DSA – digital subtraction angiography, GA – general anesthesia, ICA – internal carotid artery, ICH – intracranial hemorrhage, MCA – middle cerebral artery, mRS – modified Rankin scale, NIHSS – National Institutes of Health Stroke Scale, TIA – transient ischemic attack, TICI – Thrombolysis in Cerebral Infarction
