# Supplementary material for: Distance to thrombus, ischemic lesion volume and clinical outcome after thrombectomy for M1 middle cerebral artery occlusion
Source: Wien Klin Wochenschr. 2024 May 15;137(5-6):163–71. doi: 10.1007/s00508-024-02364-y (PMC11926011; doi:10.1007/s00508-024-02364-y)
Supplement: Supplementary file 2 — Suppl. Tab. 2 Univariate associations with clinical outcome at 3 months as measured with modified Rankin Scale (mRS). [file 508_2024_2364_MOESM2_ESM.docx]

| Variables | All patients, N = 282 | 0-2 , N =139 | 3-6 , N =143 | P value |
| --- | --- | --- | --- | --- |
| Age (years) | 76.0 (63.0-83.0) | 71.0 (60.5-79.7) | 79.0 (70.0-84.9) | <0.001 |
| Taking Vitamin-K OAC | 12 (4.3) | 2 (1.4) | 10 (7.0) | 0.035 |
| Wake-up stroke | 56 (19.9) | 21 (15.1) | 35 (24.5) | 0.053 |
| NIHSS at admission | 16.0 (12.0-19.0) | 14.0 (10.0-18.0) | 17.0 (15.0-20.0) | <0.001 |
| Systemic thrombolysis | 152 (53.9) | 85 (61.2) | 67 (46.9) | 0.017 |
|  |  |  |  |  |
| ASPECTS (<=6) | 34 (12.2) | 10 (7.3) | 24 (17.0) | 0.017 |
| Leptomeningeal collaterals (absent) | 125 (44.8) | 54 (38.8) | 71 (50.7) | 0.054 |
| Ipsilateral ICA diameter (mm) | 2.9 (2.6-3.3) | 2.9 (2.6-3.2) | 3.0 (2.7-3.3) | 0.095 |
|  |  |  |  |  |
| Thrombolysis to groin puncture (min) | 95.5 (47.0-125.0) | 84.0 (43.5-115.5) | 109.0 (51.8-128.8) | 0.027 |
| Time from groin puncture to target vessel (min) | 8.0 (5.0-15.2) | 8.0 (4.0-14.0) | 9.0 (6.0-16.0) | 0.089 |
| Total thrombectomy steps performed (N) | 1.0 (1.0-3.0) | 1.0 (1.0-2.0) | 2.0 (1.0-3.0) | 0.007 |
| First pass successful | 132 (48.2) | 80 (58.8) | 52 (37.7) | <0.001 |
| TICI outcome (2b-3) | 254 (90.1) | 131 (94.2) | 123 (86.0) | 0.027 |
| Vessel perforation | 12 (4.3) | 2 (1.4) | 10 (7.0) | 0.035 |
| Hemorrhagic transformation |  |  |  | 0.057 |
| (class 1a-c) | 61 (21.6) | 25 (18.0) | 36 (25.2) |  |
| (class 2) | 17 (6.0) | 6 (4.3) | 11 (7.7) |  |
| (class 3a-d) | 19 (6.7) | 6 (4.3) | 13 (9.1) |  |
| (no bleeding) | 185 (65.6) | 102 (73.4) | 83 (58.0) |  |
| ILV (continuous) | 11.4 (2.2-53.2) | 3.9 (1.0-18.1) | 35.9 (5.4-112.6) | <0.001 |
| ln-transfomed ILV | 2.5 (1.2-4.0) | 1.6 (0.7-2.9) | 3.6 (1.9-4.7) | <0.001 |
| ILV categories (ml) |  |  |  | <0.001 |
| (0 - 15) | 155 (55.0) | 96 (69.1) | 59 (41.3) |  |
| (15.1 - 70) | 68 (24.1) | 37 (26.6) | 31 (21.7) |  |
| (70.1 - 200) | 39 (13.8) | 5 (3.6) | 34 (23.8) |  |
| (> 200) | 20 (7.1) | 1 (0.7) | 19 (13.3) |  |

**Supplemental Table 2.** Univariate associations with clinical outcome at 3 months as measured with modified Rankin Scale (mRS). Missing data (mRS) = 18% (64 patients lost to follow-up).

OAC – oral anticoagulants; ILV – ischemic lesion volume; ln-ILV – log transformed ischemic lesion volume
