# Supplementary material for: Distance to thrombus, ischemic lesion volume and clinical outcome after thrombectomy for M1 middle cerebral artery occlusion
Source: Wien Klin Wochenschr. 2024 May 15;137(5-6):163–71. doi: 10.1007/s00508-024-02364-y (PMC11926011; doi:10.1007/s00508-024-02364-y)
Supplement: Supplementary file 3 — Suppl. Tab. 3 Multivariate logistic regression analysis of 242 patients, good clinical outcome was defined as modified Rankin scale 0–2 at 3 months. [file 508_2024_2364_MOESM3_ESM.docx]

| Predictors | Odds Ratios | CI | p |
| --- | --- | --- | --- |
| Age (years) | 0.94 | 0.91 – 0.96 | <0.001 |
| NIHSS at admission | 0.87 | 0.81 – 0.93 | <0.001 |
| Taking Vitamin-K OAC | 5.52 | 0.86 – 62.26 | 0.111 |
| Known time of symptom onset | 0.97 | 0.38 – 2.40 | 0.940 |
| Systemic thrombolysis | 1.29 | 0.65 – 2.54 | 0.467 |
| ASPECTS: >6 | 2.20 | 0.69 – 7.60 | 0.194 |
| Good or equal LC | 1.27 | 0.63 – 2.54 | 0.508 |
| Ipsilateral ICA diameter | 0.68 | 0.37 – 1.23 | 0.205 |
| Total thrombectomy steps performed | 1.10 | 0.84 – 1.44 | 0.488 |
| First pass successful | 2.10 | 0.90 – 4.97 | 0.088 |
| TICI 2b,3 | 1.23 | 0.31 – 5.47 | 0.776 |
| Vessel perforation | 0.30 | 0.03 – 2.22 | 0.280 |
| Hemorrhagic transformation |  |  |  |
| class 2 | 0.80 | 0.13 – 4.52 | 0.802 |
| class 3 a-d | 0.16 | 0.03 – 0.83 | 0.030 |
| no bleeding | 0.74 | 0.31 – 1.74 | 0.494 |
| Ln-transformed ILV | 0.52 | 0.40 – 0.67 | <0.001 |

Observations: 242

R2 Tjur 0.387

**Supplemental Table 3.** Multivariate logistic regression analysis of 242 patients, good clinical outcome was defined as modified Rankin scale 0-2 at 3 months.

Abbreviations: LC – leptomeningeal collaterals; TICI – thrombolysis in cerebral infarction; ILV – ischemic lesion volume
