# Supplementary figures and images for: Distance to thrombus, ischemic lesion volume and clinical outcome after thrombectomy for M1 middle cerebral artery occlusion
Source: Wien Klin Wochenschr. 2024 May 15;137(5-6):163–71. doi: 10.1007/s00508-024-02364-y (PMC11926011; doi:10.1007/s00508-024-02364-y)

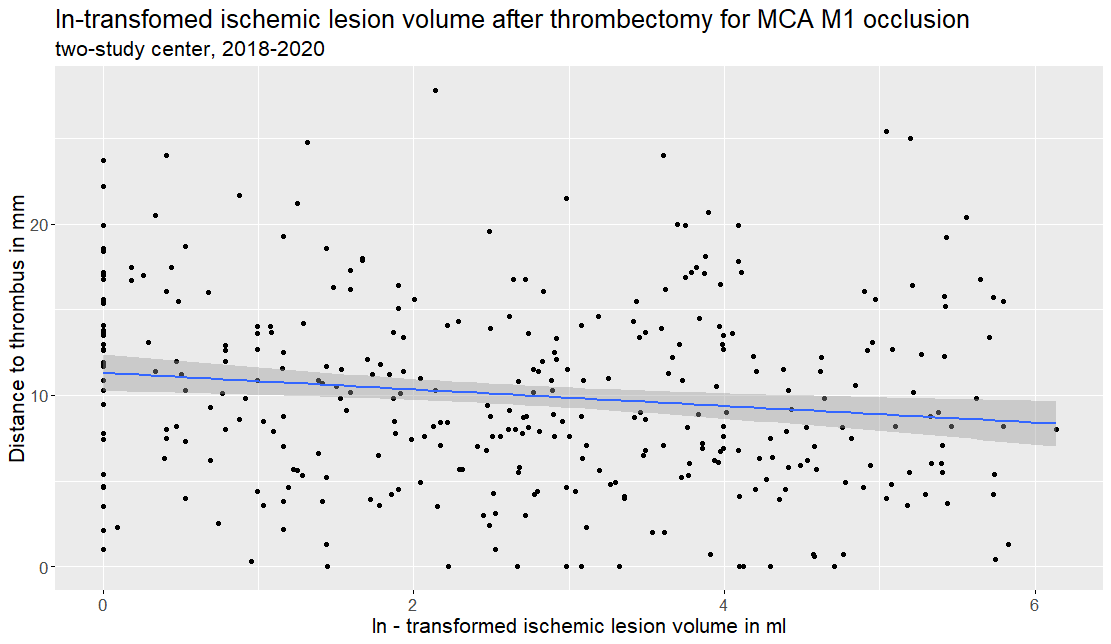

Supplement: Supplementary file 4 — Suppl. Fig. 1 ln-transformed ischemic lesion volume after thrombectomy for MCA M1 occlusion [file 508_2024_2364_MOESM4_ESM.tiff]
